# The Impact of Early In-Hospital Use of SGLT2 Inhibitors on Outcomes in Patients With Acute Heart Failure: An Updated Systematic Review and Meta-Analysis

**DOI:** 10.31083/RCM45590

**Published:** 2026-03-20

**Authors:** Yifan Deng, Yue Ma, Hao Li, Li Zhu

**Affiliations:** ^1^Medical College of Yangzhou University, 225001 Yangzhou, Jiangsu, China; ^2^Department of Cardiology, The Affiliated Taizhou People’s Hospital of Nanjing Medical University, 225300 Taizhou, Jiangsu, China; ^3^Department of General Surgery, The Hanjiang District Maternal and Child Health Care Hospital, 225100 Yangzhou, Jiangsu, China; ^4^Hubei University of Medicine, 442000 Shiyan, Hubei, China

**Keywords:** sodium-glucose cotransporter-2 inhibitors, acute heart failure, heart failure, prognosis

## Abstract

**Background:**

Sodium–glucose cotransporter 2 (SGLT2) inhibitors, employed as antidiabetic agents, have been shown to effectively improve the prognosis of patients with chronic and stable heart failure, chronic kidney disease, and diabetes in the context of cardiovascular–renal–endocrine integrated management. However, the safety and clinical benefits of the early application of SGLT2 inhibitors in hospitalized patients with acute heart failure remain controversial. This study aimed to evaluate the safety and prognostic impact of early SGLT2 inhibitor therapy in patients with acute heart failure.

**Methods:**

A systematic literature search of the PubMed, Web of Science, and Cochrane Library databases was conducted to identify studies on the use of SGLT2 inhibitors in acute heart failure. Two researchers independently screened studies, extracted data, and assessed the risk of bias in the included studies. The meta-analysis was performed using STATA 16.0 software (StataCorp, College Station, TX, USA).

**Results:**

A total of 23 studies involving 47,291 patients with acute heart failure were included in this analysis (10 randomized controlled trials and 13 observational studies). Early use of SGLT2 inhibitors in hospitalized patients with acute heart failure was associated with a reduction in the incidence of composite events in the short term (relative risk (RR) = 0.64, 95% confidence interval (CI) (0.56, 0.74)), all-cause mortality (RR = 0.72, 95% CI (0.60, 0.86)), and heart failure rehospitalization rates (RR= 0.77, 95% CI (0.63, 0.87)); however, the early use of SGLT2i did not improve the incidence of cardiogenic death (RR = 0.74, 95% CI (0.51, 1.08)). Additionally, the early administration of SGLT2 inhibitors significantly reduced the incidence of cardiogenic mortality (RR = 0.77, 95% CI (0.60, 1.0); *p* = 0.045), as well as decreasing heart failure rehospitalization rates (RR = 0.77, 95% CI (0.70, 0.86)) and all-cause mortality (RR = 0.49, 95% CI (0.41, 0.60)), without increasing the incidence of adverse drug reactions such as acute kidney injury, urinary tract infections, diabetic ketoacidosis, hypoglycemia, or hypotension.

**Conclusion:**

Early in-hospital use of SGLT2 inhibitors can safely and effectively reduce the incidence of all-cause mortality, cardiogenic rehospitalization, and composite events in acute heart failure patients in both the short term and over one year.

## 1. Introduction

Acute heart failure (AHF) is a clinical syndrome characterized by a sudden onset or exacerbation of left ventricular dysfunction, leading to decreased myocardial contractility and increased cardiac load, resulting in a rapid decline in acute cardiac output [1]. Globally, the prevalence of heart failure (HF) continues to rise and has become one of the most common causes of hospitalization in individuals aged 65 and older. Despite modern treatment options, the in-hospital mortality rate for AHF patients remains at 8–10%, with a 30-day readmission rate reaching 25%, thus posing a significant public health burden [2]. Therefore, recent research has focused on multi-target drugs that can effectively improve prognosis. Sodium-glucose co-transporter 2 inhibitors (SGLT2i) are a novel class of oral hypoglycemic agents that primarily target the SGLT2 receptors in the renal proximal tubules, inhibiting glucose reabsorption [3]. Recent clinical randomized trials have demonstrated that SGLT2i have beneficial effects in cardiovascular and renal diseases, and this effect extends to non-diabetic populations. The 2023 European Society of Cardiology (ESC) guidelines included SGLT2i as one of the four cornerstone therapies for heart failure with reduced ejection fraction (HFrEF (Heart Failure with Reduced Ejection Fraction), left ventricular ejection fraction (LVEF) ≤40%), alongside angiotensin receptor–neprilysin inhibitor (ARNI)/angiotensin-converting enzyme inhibitors (ACEI), β-blockers, and mineralocorticoid receptor antagonists (MRA), forming the “new quadruple therapy”. Furthermore, SGLT2i is the only drug currently proven to have prognostic benefits in heart failure with preserved ejection fraction (HFpEF) [4]. Some researchers have shifted the focus of SGLT2i to AHF, although the timing of its application and its corresponding clinical benefits remain controversial. Existing meta-analyses have not provided a comprehensive analysis of clinical benefits at different time points, nor a detailed evaluation of drug safety [5]. Therefore, this study includes 22 studies to investigate the safety and clinical benefits of early SGLT2i application in AHF patients, aiming to provide new insights and evidence-based medicine for the use of SGLT2i in AHF.

## 2. Methods

This study was designed and conducted under the Preferred Reporting Items for Systematic Review and Meta-Analysis (PRISMA) guidelines for the systematic evaluation and Meta-analysis.

### 2.1 Literature Search

Using English keywords and their synonyms (acute heart failure) AND (Sodium glucose transporter 2 inhibitor OR SGLT-2 inhibitor) for a combined search in PubMed, Web of Science, and Cochrane Library, limited to English-language literature, with the search period from October 2013 to June 2025. Secondary screening of the literature will be performed through reading the articles.

### 2.2 Inclusion and Exclusion Criteria

#### 2.2.1 Inclusion Criteria

(1) Randomized controlled trials or retrospective studies. (2) Study population consisting of patients diagnosed with acute heart failure who require pharmacological treatment. The control group should consist of patients receiving conventional drug therapy/SLGT-2 inhibitor therapy after discharge/placebo therapy, while the study group should involve patients who start using SGLT-2 inhibitors upon hospitalization. (3) Studies must provide clear information on patient demographics, follow-up duration, and related endpoint events.

#### 2.2.2 Exclusion Criteria

(1) Sample size (patients receiving SGLT-2 inhibitors) less than 10. (2) Duplicate publications. (3) Article types: conference abstracts, reviews, commentaries, case reports, or studies involving cells or animals. (4) Studies that do not provide detailed information about the study population, endpoint event definitions, and major adverse cardiovascular events (MACEs) occurrence, and where contacting the authors for further information is not possible.

### 2.3 Data Extraction

Two researchers will independently extract data from studies that meet the inclusion and exclusion criteria, then cross-check the extracted information. The data to be extracted includes:

(1) Basic study information: first author, publication year, inclusion criteria, etc. (2) Basic information on enrolled patients: number of patients, age, gender, relevant medical history, follow-up duration, etc. (3) Outcome measures: short-term/medium- to long-term major adverse cardiovascular events (MACEs) and drug-related adverse events timeline. (4) Bias risk assessment-related elements.

### 2.4 Statistical Methods

Stata 16.0 software (StataCorp, College Station, TX, USA) will be used to conduct a meta-analysis of the length of hospital stay, short-term/medium- to long-term major cardiovascular adverse events, and drug-related adverse event timelines for studies included. Relative risk (RR) will be used to assess the differences in the occurrence of clinical events between different treatment regimens. I^2^ statistics will be used to assess the heterogeneity of the studies. I^2^ values of 75%, 50%, and 25% represent high, medium, and low heterogeneity, respectively. If there is no statistical heterogeneity between the studies, a fixed-effect model will be used for meta-analysis. If heterogeneity exists, subgroup analysis, sensitivity analysis, and trim-and-fill method will be used to further explore the sources of heterogeneity. Funnel plots and Egger’s test will be used to assess publication bias. A *p*-value of <0.05 will be considered statistically significant.

## 3. Results

### 3.1 Inclusion of Literature

**Fig. 1.**
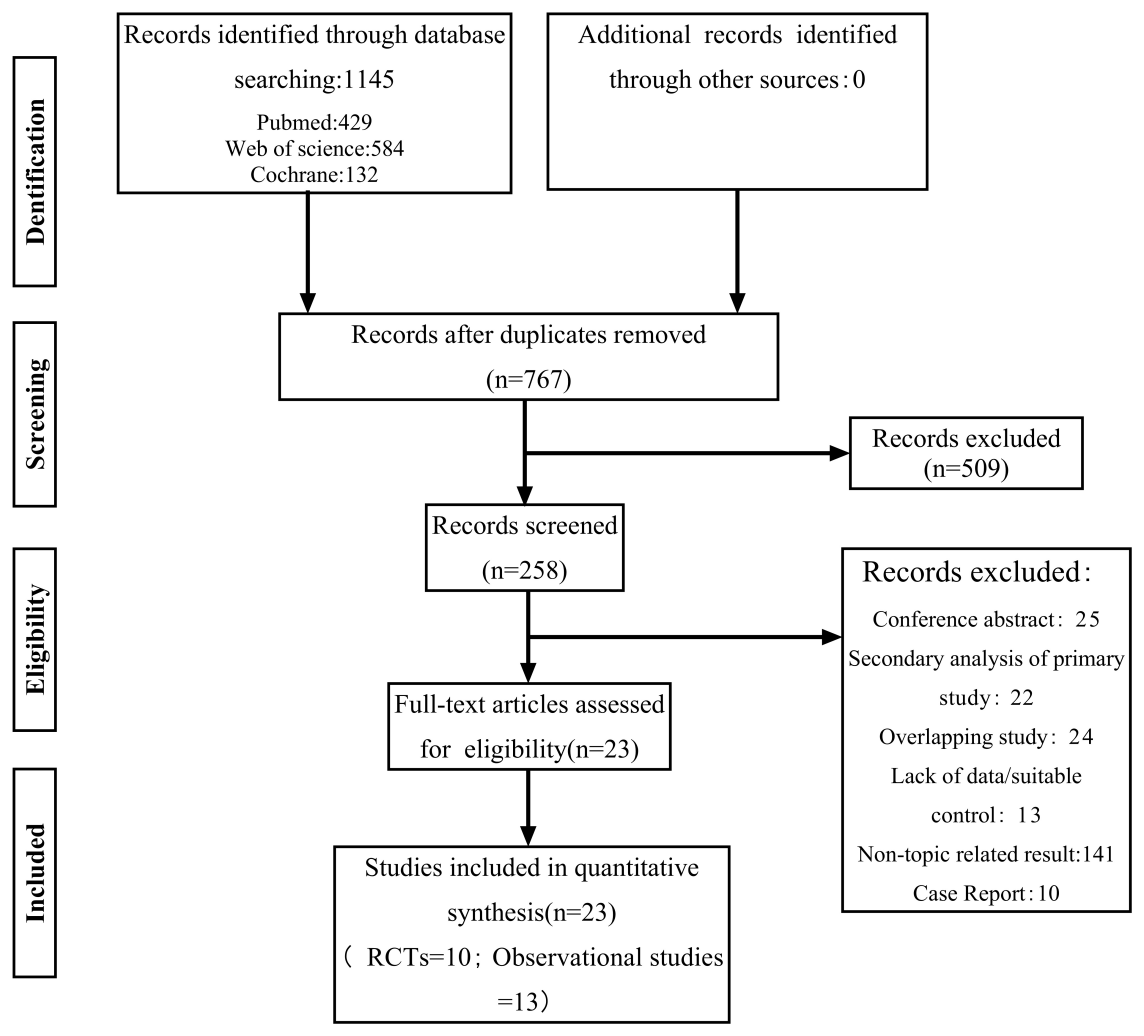
**Study screening procedures and outcomes**. RCTs, randomized controlled trials.

Literature inclusion status: A total of 1145 articles were retrieved, including 429 from PubMed, 584 from Web of Science, and 132 from Cochrane Library. After excluding 378 duplicate articles, 258 articles were preliminarily screened by reading the titles and abstracts. Following a detailed review of the full texts of the initially screened articles, 23 articles were ultimately included. Among these, 13 observational studies [6, 7, 8, 9, 10, 11, 12, 13, 14, 15, 16, 17, 18] and 10 randomized controlled trials [19, 20, 21, 22, 23, 24, 25, 26, 27, 28] were selected. The specific search process is shown in Fig. 1. The evaluation process was carried out by two researchers separately and cross-checked. The bias in the included articles was evaluated using the quality assessment criteria from the Cochrane Handbook and The Newcastle-Ottawa Scale, as seen in the **Supplementary Table 1** and **Supplementary Fig. 1**.

### 3.2 Baseline Characteristics of Included Studies

This study included 23 studies involving a total of 47,261 acute heart failure (AHF) patients, among whom 7294 received early SGLT2 inhibitor (SGLT2i) treatment, and 39,967 received standard heart failure treatment, delayed SGLT2i, or placebo. The baseline characteristics of all included patients are shown in Table 1 (Ref. [6, 7, 8, 9, 10, 11, 12, 13, 14, 15, 16, 17, 18, 19, 20, 21, 22, 23, 24, 25, 26, 27, 28]).

**Table 1.** **Study Characteristics**.

Author	Year	Sample size		Age		Male		Diabetes mellitus		NYHA functional class		Duration of SGLT2i use and dosage
		(N (%))				N (%)		N (%)		III-IV		
		SGLT2	NO-SGLT2	SGLT2	NO-SGLT2	SGLT2	NO-SGLT2	SGLT2	NO-SGLT2	SGLT2	NO-SGLT2	SGLT2
Llorens P *et al*. [6]	2025	366	2693	81 (72−88)	86 (79−90)	193 (52.7)	1085 (40.3)	178 (49.6)	931 (34.9)	50 (16.5)	428 (18.6)	before discharge
Park S *et al*. [7]	2023	818	28,472	69.1 (12.2)	74.2 (10.7)	406 (49.6)	12,445 (43.7)	all	all	225 (27.5)	9437 (33.1)	before discharge
Pérez-Belmonte LM *et al*. [8]	2022	99	109	84.5 ± 4.0	86.4 ± 4.8	54 (54.5)	50 (45.9)	all	all	40	41	before discharge
Matsukawa R *et al*. [9]	2023	92	76	71.4 ± 13.3	72.0 ± 14.2	66 (71.7)	53 (69.7)	all	all	75	67	within 48 hours of admission
Nakagaito M *et al*. [10]	2021	56	30	69.6 ± 11.5	75.8 ± 13.0	38 (68)	17 (57)	all	all	/	/	before discharge
Echeverría LE *et al*. [11]	2025	1275	395	68.0 (59.0–76.0)	69.0 (58.5–77.0)	384 (30.1)	84 (24.3)	222 (17.4)	73 (18.5)	855 (67.1)	237 (60.0)	within 48 hours of admission
Calcagno T *et al*. [12]	2025	2043	2043	68.1 ± 11.0	68.5 ± 11.0	928 (45.4)	929 (45.5)	1649 (80.7)	1661 (81.3)			before discharge
Kambara T *et al*. [13]	2019	12	19	73 ± 9	75 ± 10	9 (75)	14 (73)	all	all	10	17	within 24 hours of admission
Burgos LM *et al*. [14]	2024	237	141	/	/	/	/	/	/	/	/	before discharge
Amioka M *et al*. [15]	2025	163	198	82.5 ± 4.6	83.3 ± 4.6	90 (55.2)	103 (52)	68 (41.7)	71 (35.9)	79 (48.5)	98 (49.5)	within 48 hours of admission
Aklilu AM *et al*. [16]	2023	356	2949	69.5 (60.4–78.5)	78.7 (68.1–87.3)	219 (61.5)	1428 (48.4)	241 (67.7)	1470 (49.8)	/	/	within 10 days of admission
Guzmán-Carreras A *et al*. [17]	2024	210	540	82 ± 2	84 ± 9	102 (48.6)	215 (39.8)	132 (62.9)	213 (39.4%)	91	212	before discharge
Wu D *et al*. [18]	2024	206	193	75.00 (60.75, 79.25)	75.00 (67.00, 82.00)	102 (49.51)	96 (49.74)	102 (49.51)	89 (46.11)	160	180	within 6 days of admission
El-Gazar RA *et al*. [19]	2025	71	71	55 (40–63)	56 (48–61)	54 (76.1)	48 (67.6)	30 (42.3)	35 (49.3)	70	67	within 24 hours of admission; 100 mg/day of canagliflozin
Emara AN *et al*. [20]	2023	45	42	63.9 (10)	61.1 (11.8)	35 (77.8)	27 (64.3)	16 (35.6)	22 (52.4)	/	/	
Cox ZL *et al*. [21]	2024	119	119	65 (56–73)	64 (55–74)	78 (66)	67 (56)	84 (71)	85 (71)	/	/	within 24 hours of admission; Dapagliflozin 10 mg/day
Schulze PC *et al*. [22]	2022	30	29	72.9 ± 11.2	76.5 ± 8.3	19 (63.3)	17 (58.6)	13/30 (43.3)	10/29 (34.5)	27	24	within 24 hours of admission; Empagliflozin 10 mg/day
Damman K *et al*. [23]	2020	40	39	79 (73–83)	73 (61–83)	24 (60)	29 (74.4)	38	28	92	97	within 24 hours of admission; Empagliflozin 10 mg/day
Voors AA *et al*. [24]	2022	265	265	71 (62–78)	70 (59–78)	179 (67.5)	172 (64.9)	124 (46.8)	116 (43.8)	160	168	within 24 hours of admission; Empagliflozin 10 mg/day
Charaya K *et al*. [25]	2022	50	52	72.6 ± 12.2	74.2 ± 11.3	29 (58)	27 (52)	15 (30)	16 (30)	16 (34)	23 (44)	within 24 hours of admission; Empagliflozin 10 mg/day
Ibrahim A *et al*. [26]	2020	50	50	62.02 ± 8.8	60.64 ± 9.9	28 (56)	26 (52)	all	all	/	/	within 24 hours of admission; Dapagliflozin 10 mg/day
Bhatt DL *et al*. [27]	2021	608	614	69 (63–76)	70 (64–76)	410 (67.4)	400 (65.1)	/	/	/	/	within 3 days before or after discharge; sotagliflozin 200 mg/day
López-Vilella R *et al*. [28]	2022	83	453	72.4 ± 12.6	73.4 ± 12.6	53	256	54	140	13	72	before discharge

SGLT2, Sodium–glucose cotransporter 2; NYHA, New York Heart Association.

### 3.3 Short-Term Prognostic Impact

**Fig. 2.**
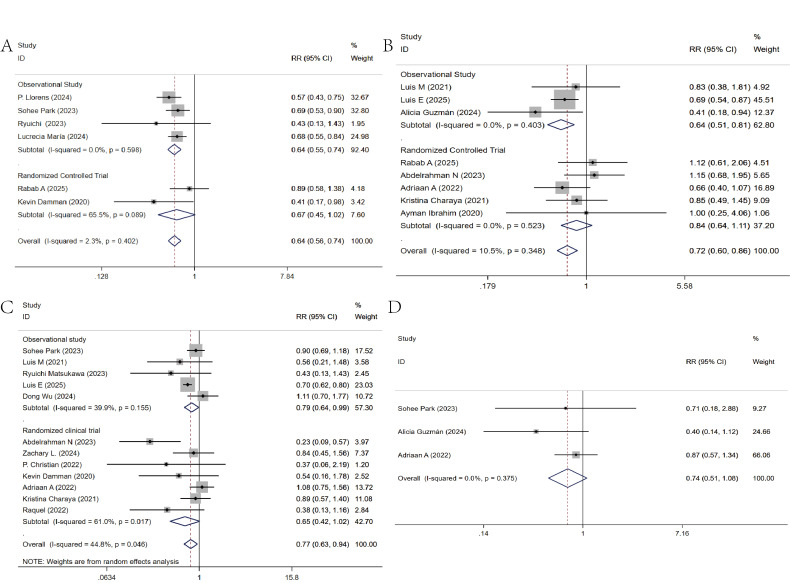
**Meta-analysis of short-term prognostic impacts**. (A) Composite event rates. (B) All-cause mortality. (C) Heart failure rehospitalization rates. (D) Cardiovascular mortality. CI, confidence interval; RR, relative risk.

The short-term composite event incidence was included in 7 studies [6, 7, 8, 9, 14, 19, 23]. These studies exhibited high heterogeneity (I^2^ = 78.6%). Through sensitivity analysis, we found that the study by Pérez-Belmonte LM [8] had a potential risk of bias (**Supplementary Fig. 2**). After excluding this study, we found that early use of SGLT2 inhibitors could reduce the incidence of short-term composite events in patients [RR = 0.64, 95% CI (0.56, 0.74)] (Fig. 2A).

The all-cause mortality rate in the short term was included in 13 studies [8, 11, 16, 17, 18, 19, 20, 21, 22, 23, 24, 25, 26]. These studies exhibited high heterogeneity (I^2^ = 83.4%). Through sensitivity analysis, we found that the studies by DONG Wu [18] and Aklilu AM [16] had significant risk of bias (**Supplementary Fig. 3**). After excluding these two studies, we found that early use of SGLT2 inhibitors could reduce the all-cause mortality rate in the short term [RR = 0.72, 95% CI (0.60, 0.86)] (Fig. 2B).

The incidence of heart failure-related rehospitalization in the short term was included in 12 studies [7, 8, 9, 11, 18, 20, 21, 22, 23, 24, 25, 28]. These studies exhibited moderate heterogeneity (I^2^ = 44.8%). Using a random-effects analysis, we found that early use of SGLT2 inhibitors could reduce the incidence of heart failure-related rehospitalization in the short term [RR = 0.77, 95% CI (0.63, 0.94)] (Fig. 2C).

The short-term cardiogenic mortality rate was included in three studies [7, 17, 24], and there was no heterogeneity (I^2^ = 0%). Fixed-effect analysis showed that early use of SGLT2 inhibitors did not affect short-term cardiogenic mortality [RR = 0.74, 95% CI (0.51, 1.08), *p* = 0.121] (Fig. 2D).

The specific analysis results are shown in Table 2.

**Table 2.** **Meta-analysis of short-term prognostic impacts**.

	Heterogeneity test		META analysis		
	I^2^	*p*	RR 95% CI	Z	*p*
Composite event rates	2.3%	0.402	0.64 (0.56, 0.74)	6.2	<0.001
All-cause mortality	10.5%	0.348	0.72 (0.60, 0.86)	3.66	<0.001
Heart failure rehospitalization rates	44.8%	0.046	0.77 (0.63, 0.94)	2.54	0.011
Cardiovascular mortality	0%	0.375	0.74 (0.51, 1.08)	1.55	0.121

### 3.4 One-Year Prognostic Impact

**Fig. 3.**
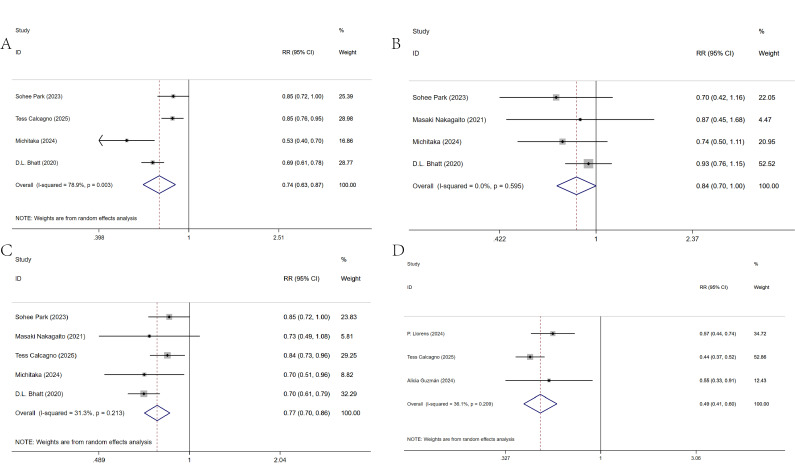
**Meta-analysis of one-year prognostic impact**. (A) Composite event rates. (B) Cardiovascular mortality. (C) Heart failure rehospitalization rates. (D) All-cause mortality.

For the incidence of composite events within 1 year, four studies were included [7, 12, 15, 27], with significant heterogeneity among the studies (I^2^ = 78.9%, *p* = 0.003). Sensitivity analysis showed no significant reduction in heterogeneity after excluding any single study (**Supplementary Fig. 4**). A random-effects model analysis revealed that early use of SGLT2 inhibitors significantly reduced the incidence of composite events within 1 year in patients [RR = 0.74, 95% CI (0.63, 0.87)], (Fig. 3A).

For the incidence of cardiogenic mortality within 1 year, four studies were included [7, 10, 15, 27], with no heterogeneity (I^2^ = 0%). A fixed-effects model analysis showed that early use of SGLT2 inhibitors significantly reduced the cardiogenic mortality rate within 1 year [RR = 0.84, 95% CI (0.70, 0.99)], (Fig. 3B).

For the heart failure rehospitalization rate within 1 year, five studies were included [7, 10, 12, 15, 27], with moderate heterogeneity among the studies (I^2^ = 31.3%). A random-effects model analysis revealed that early use of SGLT2 inhibitors significantly reduced the heart failure rehospitalization rate within 1 year [RR = 0.77, 95% CI (0.70, 0.86)], (Fig. 3C).

For all-cause mortality within 1 year, three studies were included [6, 12, 17], with significant heterogeneity among the studies (I^2^ = 36.1%). A random-effects model analysis showed that early use of SGLT2 inhibitors significantly reduced all-cause mortality within 1 year [RR = 0.49, 95% CI (0.41, 0.60)], (Fig. 3D).

The specific analysis results are shown in Table 3.

**Table 3.** **Meta-analysis of one-year prognostic impact**.

	Heterogeneity test		META analysis		
	I^2^	*p*	RR 95% CI	Z	*p*
Cardiovascular mortality	0%	0.595	0.84 (0.70, 1.0)	1.95	0.05
Heart failure rehospitalization rates	31.3%	0.393	0.77 (0.70, 0.86)	4.99	<0.001
All-cause mortality	36.1%	0.209	0.49 (0.41, 0.60)	7.14	<0.001

### 3.5 Safety of Drug Use

**Fig. 4.**
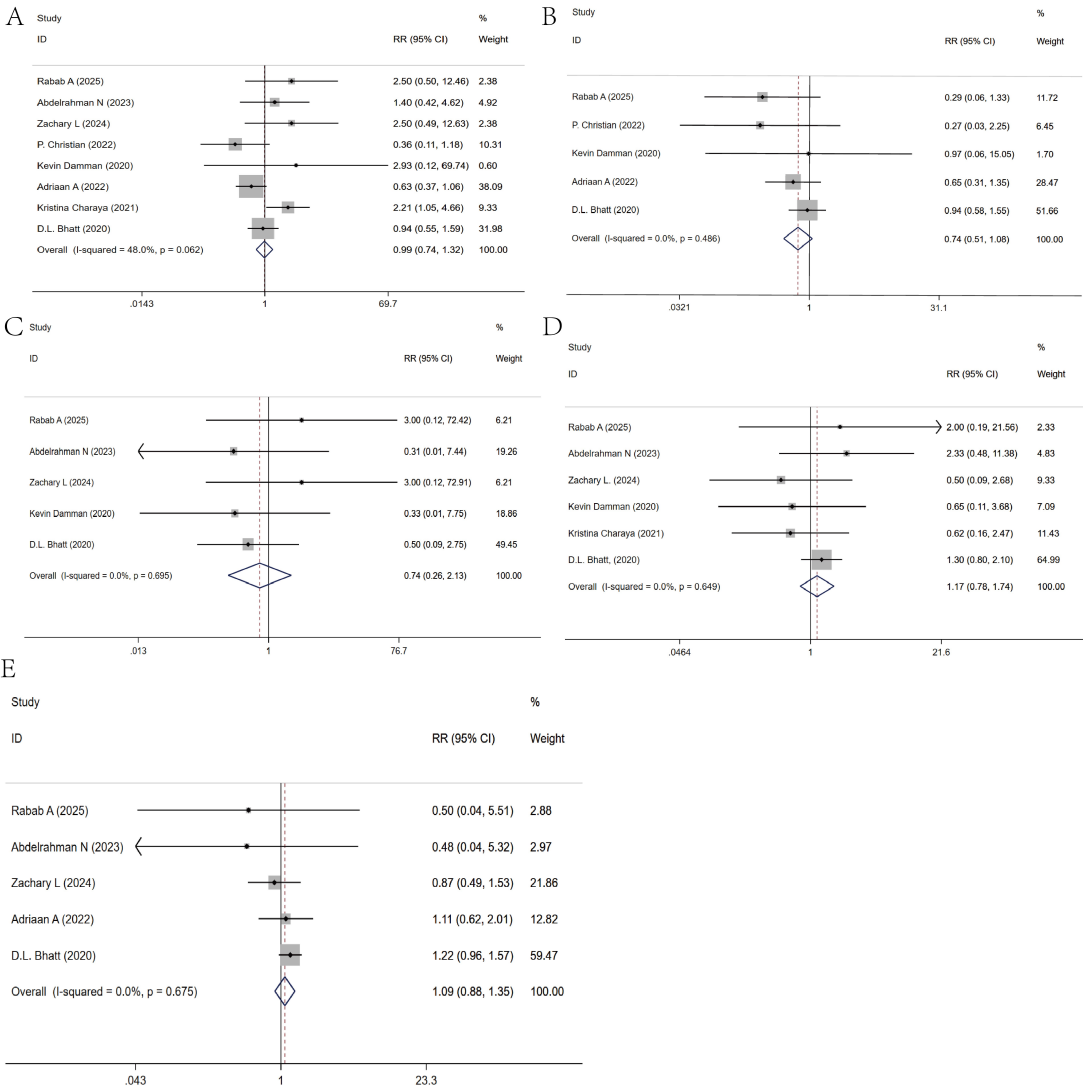
**Meta-analysis of drug safety**. (A) Acute kidney injury. (B) Urinary tract infections. (C) Ketoacidosis. (D) Hypotension. (E) Hypoglycemia.

Regarding the safety of SGLT2i in AHF patients, we compared the most common adverse drug events and found that SGLT2i did not increase the incidence of acute kidney injury [19, 20, 21, 22, 23, 24, 25, 27], (Fig. 4A), urinary tract infections [19, 22, 23, 24, 27], (Fig. 4B), ketoacidosis [20, 21, 22, 23, 28], (Fig. 4C), hypotension [19, 20, 21, 22, 25, 27], (Fig. 4D), or hypoglycemia [19, 20, 21, 24, 27], (Fig. 4E) (Table 4).

**Table 4.** **Meta-analysis of drug safety**.

	Heterogeneity test		META analysis		
	I^2^	*p*	RR 95% CI	Z	*p*
Acute kidney injury	48%	0.062	0.99 (0.74, 1.32)	0.1	0.924
Urinary tract infections	0%	0.468	0.74 (0.51, 1.08)	1.56	0.119
Ketoacidosis	0%	0.695	0.74 (0.26, 2.13)	0.55	0.581
Hypotension	0%	0.649	1.17 (0.78, 1.74)	0.76	0.446
Hypoglycemia	0%	0.723	1.08 (0.88, 1.35)	0.77	0.441

### 3.6 Bias Assessment

Bias assessment of the above results indicated a low likelihood of bias, confirming the robustness of the meta-analysis findings. See **Supplementary Figs. 5,6**.

## 4. Discussion

This may be the largest meta-analysis and systematic review to date in terms of the number of studies included and sample size. It comprehensively explores and demonstrates that the early use of SGLT2i in-hospital can safely and effectively reduce the incidence of composite events, such as all-cause mortality and cardiogenic rehospitalization, both in the short term and within 1 year, in patients with acute heart failure (AHF). This suggests potential benefits in improving patient prognosis.

Initially, SGLT2i were introduced as a treatment for diabetes. However, with the emergence of numerous randomized controlled trials and evidence from evidence-based medicine, the clinical indications for SGLT2i have expanded to include non-diabetic populations and other diseases. The DELIVER study highlighted that SGLT2i significantly reduced the risk of major composite outcomes in patients with chronic heart failure (HF), including 18% of those with HF with improved ejection fraction (HFimpEF) [HR 0.74, 95% CI (0.41–0.96)], and reduced the risk of the first heart failure worsening event by 22% [HR 0.78, 95% CI (0.61–1.14)] [29]. Following this, some researchers hypothesized that SGLT2i might also provide clinical benefits in AHF. The EMPULSE study was the first to confirm this hypothesis, showing that, compared to placebo, administration of dapagliflozin 10 mg within 3 days of hospitalization for newly diagnosed AHF significantly improved 90-day clinical outcomes in AHF patients (stratified win ratio: 1.36; 95% CI: 1.09–1.68; *p* = 0.0054), including improvements in the Kansas City Cardiomyopathy Questionnaire (KCCQ) total symptom score, time to all-cause mortality, or first heart failure event [24]. However, the DAPA-RESPONSE-AHF study pointed out that within 24 hours of hospitalization, randomization to dapagliflozin reduced rehospitalization within 30 days after discharge, but had no impact on the incidence of heart failure worsening or mortality during hospitalization [20]. Additionally, potential adverse effects of SGLT2 inhibitors may also affect the safety of AHF patients. Due to the complex physiological status of patients in the acute phase, common side effects such as hypoglycemia, dehydration, and further renal function impairment may pose a greater risk to these patients [30]. A study by Voors AA *et al*. [31] noted that SGLT2 inhibitors may cause early renal impairment in AHF patients, though this damage was no longer significant after 90 days, with similar rates of acute kidney events between groups. However, this has led to concerns regarding the early use of SGLT2 inhibitors in AHF patients. Therefore, SGLT2 inhibitors still require more evidence from randomized controlled trials and further evidence-based studies.

A meta-analysis conducted by Carvalho PEP *et al*. [32] summarized the treatment effects of SGLT2i within 30 days of acute AHF onset. It found that compared to traditional treatment groups, SGLT2i reduced all-cause mortality (OR: 0.75; 95% CI 0.56–0.99; *p* = 0.049), heart failure (HF) readmission (OR: 0.54; 95% CI 0.44–0.66; *p* < 0.001), and the composite of cardiovascular death and HF readmission (RR: 0.71; 95% CI 0.60–0.84; *p* < 0.001). In another meta-analysis by Hou J *et al*. [5], SGLT2i was shown to reduce the number of short-term HF deterioration events, hospital readmissions due to HF, and improve quality of life in AHF patients (standardized mean difference (SMD) = –0.24, 95% CI: –0.40 to –0.09, *p* = 0.002), without increasing the incidence of adverse events (RR = 0.91, 95% CI: 0.82–1.01, *p* = 0.06). In the current meta-analysis, we aggregated additional literature that met our inclusion criteria to further verify and expand upon these findings. First, we compared the incidence of MACEs within 30 days, including composite event rates, all-cause mortality, and cardiovascular readmission. We found that early use of SGLT2i provided benefits to patients, consistent with previous studies. However, no significant difference was observed in the incidence of cardiogenic death within 30 days between the SGLT2i and control groups, likely due to the short duration of drug use or the severity of AHF. Subsequently, we compared the overall event rates within one year and found that clinical benefits were sustained through the entire year post-discharge, including reduced cardiogenic mortality, HF readmission, and all-cause mortality. Although the composite event rate within one year was lower in the SGLT2i group compared to the control group, we consider this as a major result, accounting for the high heterogeneity among the included studies. This may also be the first meta-analysis comparing the short- and long-term effects of SGLT2i on AHF patients. Furthermore, our study compared the incidence of adverse drug events during SGLT2i use: common adverse effects such as acute kidney injury (AKI), urinary tract infections, hypoglycemia, and hypotension showed no significant differences between the placebo/control group and SGLT2i group, which is one of the novel aspects of this research.

Although existing evidence confirms that early use of SGLT2i can safely and effectively yield clinical benefits for AHF patients in the short and long term, some issues remain unresolved. First, the mechanisms underlying these benefits are still unclear. Kasperova BJ *et al*. [33] have pointed out that SGLT-2 inhibitors significantly downregulate pro-inflammatory gene expression in epicardial adipose tissue (EAT) and reduce macrophage and T lymphocyte infiltration in EAT, thereby decreasing oxidative stress and ferroptosis, which protects the myocardium. However, the protective mechanism of SGLT2i in AHF remains uncertain. Second, the optimal timing for early use of SGLT2i is still undefined. Research by Echeverría LE *et al*. [11] has suggested that administering SGLT2i within 48 hours of AHF hospitalization was associated with a reduction in in-hospital mortality (RR 0.37; 95% CI, 0.17–0.77), shorter hospital stay, and lower 30-day composite mortality/HF readmission rates (RR 0.72; 95% CI, 0.53–0.98). Our study mainly compared the clinical benefits of early inpatient use versus no use or post-discharge use, but the exact timing (immediately, 24 hours, or 48 hours after admission) for optimal benefit in AHF patients still requires further clinical validation. Lastly, more subgroup analyses are needed to investigate the use of SGLT2i in different populations, such as those with or without diabetes, hypertension, renal insufficiency, and gender differences. Currently, the latest guidelines for AHF recommend that SGLT2i treatment should be initiated in AHF patients either before discharge or early after discharge. Additionally, the DAPA ACT HF-TIMI 68 Trial, the largest randomized controlled trial to date, will soon release its follow-up results, and we look forward to its findings, which may provide further evidence for the early use of SGLT2i in AHF patients [34].

## 5. Limitations and Future Directions

(1) Since only one study reported the improvement of quality of life with SGLT2 inhibitors, this study did not perform a comparative analysis on the impact of early use of SGLT2 inhibitors on quality of life.

(2) Due to the differences in the definition of “early” treatment across studies, this research did not define or categorize specific time points for early treatment.

(3) Due to differences in study populations, objectives, and the lack of specific individualized data, this study only performed subgroup analysis based on study type. Future randomized controlled trials targeting specific populations, heart failure classifications, and treatment duration are anticipated.

(4) This study only included relevant English-language literature, which may have excluded studies published in other languages.

## 6. Conclusion

In summary, early use of SGLT2i effectively reduces the incidence of all-cause mortality, cardiogenic readmission, and other composite events in AHF patients over the short term and up to one year, without increasing the occurrence of adverse drug events such as hypoglycemia, hypotension, renal impairment, or ketoacidosis. However, further high-quality and large-scale clinical evidence is needed to confirm its hemodynamic effects and clinical benefits in non-diabetic populations.

## Availability of Data and Materials

All data in this study are available from the original cited studies.

## Supplementary Material

Supplementary material associated with this article can be found, in the online version, at https://doi.org/10.31083/RCM45590.
