# Supplementary material for: The Impact of Early In-Hospital Use of SGLT2 Inhibitors on Outcomes in Patients With Acute Heart Failure: An Updated Systematic Review and Meta-Analysis
Source: Rev Cardiovasc Med. 2026 Mar 20;27(3):45590. doi: 10.31083/RCM45590 (PMC13036538; doi:10.31083/RCM45590)
Supplement: Supplementary file 1 [file 2153-8174-27-3-45590-s1.zip › Supplementary Material.docx]

Supplementary Materials

Supplementary Table 1 The Newcastle-Ottawa Scale

|  |  | Selection |  |  |  | Comparability |  | Exposure |  |  | Total |
| --- | --- | --- | --- | --- | --- | --- | --- | --- | --- | --- | --- |
|  |  | Is the case definition adequate | Representativeness of the cases | Selection of Controls | Definition of Controls | study controls for the most important factor | study controls for any additional factor | Ascertainment of exposure | Same method of ascertainment for cases and controls | Non-Response rate |  |
| P. Llorens | 2024 | √ | √ | √ | √ | √ | √ | √ | √ |  | 8 |
| Sohee Park | 2023 | √ | √ | √ | √ | √ | √ | √ | √ |  | 8 |
| Luis M | 2021 | √ |  | √ | √ | √ | √ | √ | √ | √ | 8 |
| Ryuichi Matsukawa | 2023 | √ |  | √ | √ | √ | √ | √ | √ |  | 7 |
| Masaki Nakagaito | 2021 | √ |  | √ | √ | √ | √ | √ | √ |  | 7 |
| Luis E | 2025 | √ | √ | √ | √ | √ | √ | √ | √ |  | 8 |
| Tess Calcagno | 2025 | √ | √ | √ | √ | √ | √ | √ | √ | √ | 9 |
| Takahiro Kambara | 2019 | √ |  | √ | √ |  | √ | √ | √ |  | 6 |
| Lucrecia María | 2024 | √ | √ | √ | √ | √ | √ | √ | √ |  | 8 |
| Michitaka Amioka | 2024 | √ | √ | √ | √ | √ | √ | √ | √ | √ | 9 |
| Abinet M. | 2023 | √ | √ | √ | √ | √ | √ | √ | √ |  | 8 |
| Alicia Guzmán | 2024 | √ | √ | √ | √ | √ | √ | √ | √ |  | 8 |
| Dong Wu | 2024 | √ | √ | √ | √ | √ | √ | √ | √ |  | 8 |


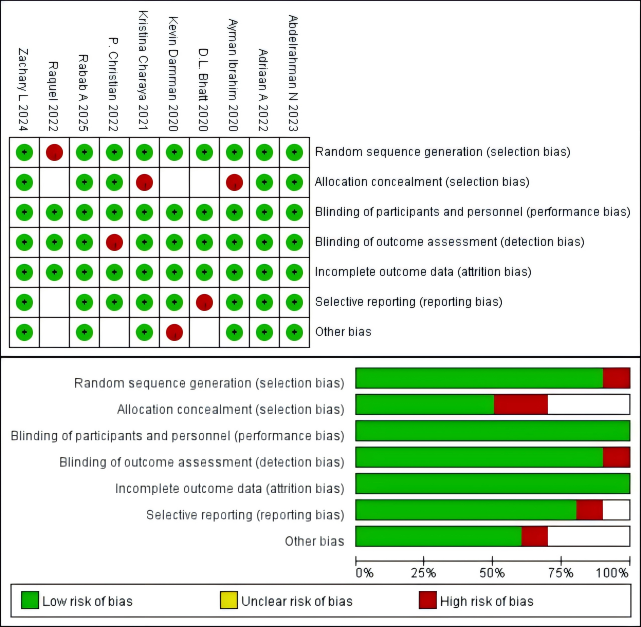


Supplementary Fig. 1 Cochrane Risk of Bias Assessment Tool


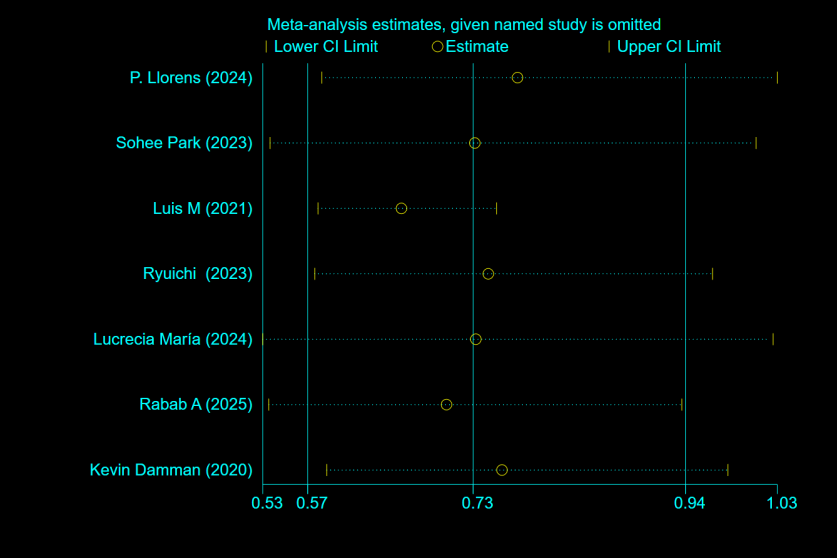


Supplementary Fig. 2 Sensitivity Analysis of the Short-term Composite Event


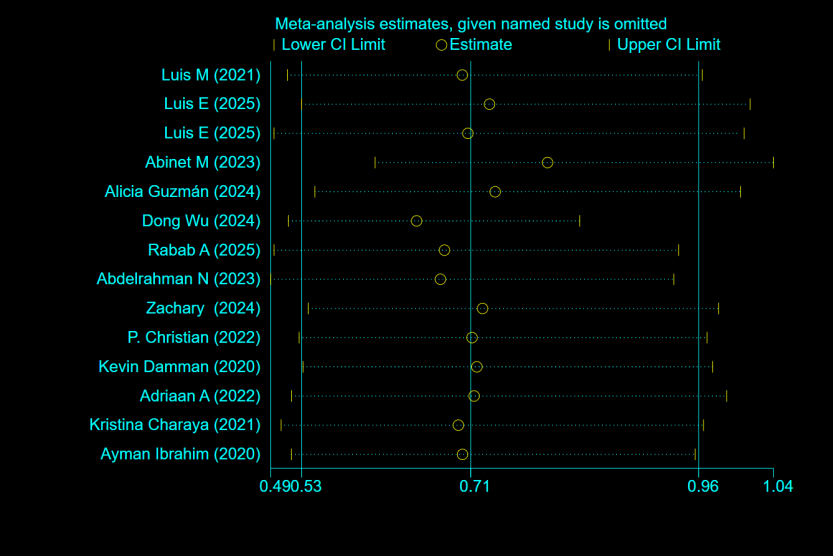


Supplementary Fig. 3 Sensitivity Analysis of the all-cause mortality rate in the short term


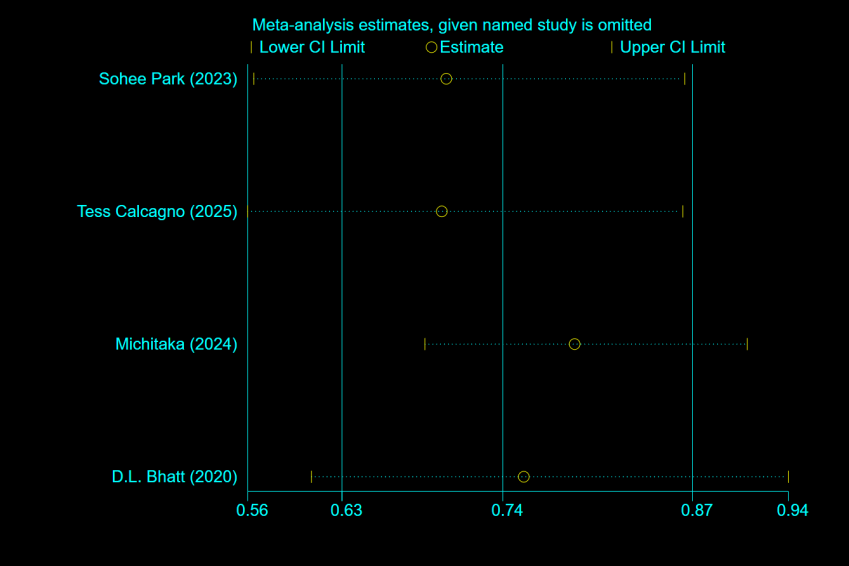


Supplementary Fig. 4 Sensitivity Analysis of the incidence of composite events within 1 year


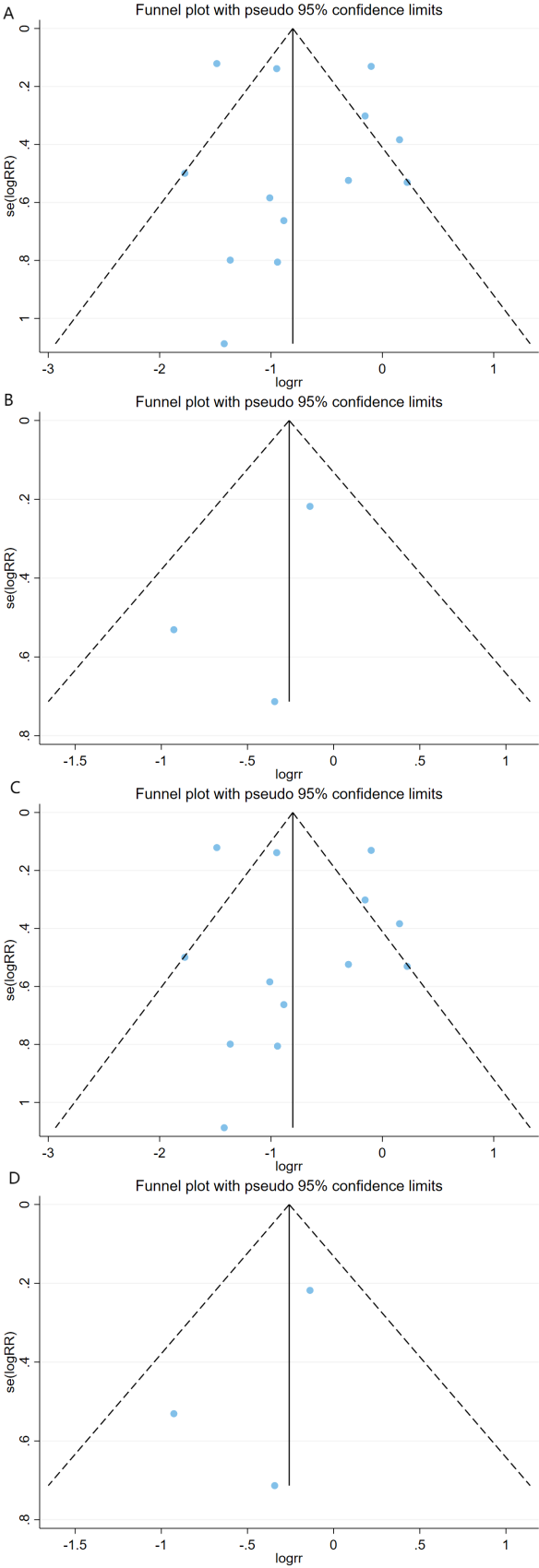


Supplementary Fig. 5 Tests for bias.A:Composite event rates;B:All-cause mortality;C:Heart failure rehospitalization rates;D:Cardiovascular mortality


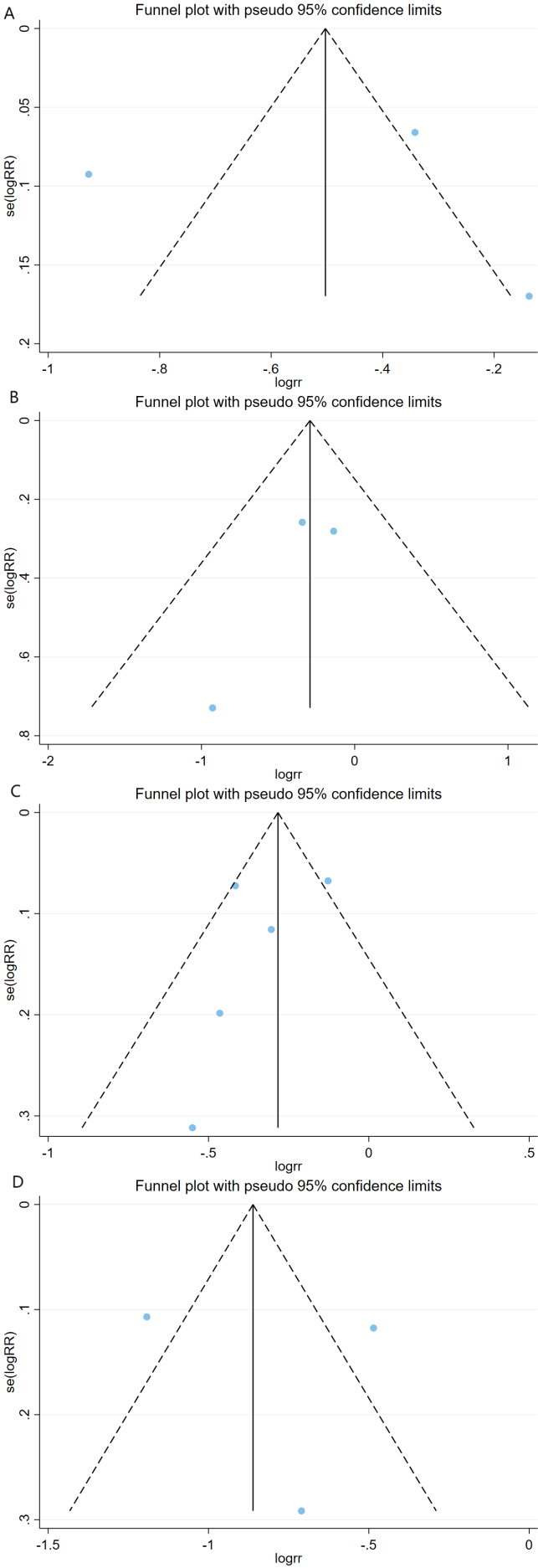


Supplementary Fig. 6 Tests for bias.A:Composite event rates;B:Cardiovascular mortality;C:Heart failure rehospitalization rates;D:All-cause mortality
